# Inhibitory Control in Aging: The Compensation-Related Utilization of Neural Circuits Hypothesis

**DOI:** 10.3389/fnagi.2021.771885

**Published:** 2022-07-28

**Authors:** Weixi Kang, Junxin Wang, Antonio Malvaso

**Affiliations:** ^1^Imperial College London, London, United Kingdom; ^2^Beijing University of Chinese Medicine, Beijing, China; ^3^School of Medicine and Surgery, Vita-Salute San Raffaele University, Milan, Italy

**Keywords:** inhibitory control, aging, cognitive decline, cognitive aging, CRUNCH, compensation-related utilization of neural circuits hypothesis

## Abstract

As one of the core executive functions, inhibitory control plays an important role in human life. Inhibitory control refers to the ability to suppress task irrelevant information both internally and externally. Modern cognitive neuroscience has extensively investigated the neural basis of inhibitory control, less is known about the inhibitory control mechanisms in aging. Growing interests in cognitive declines of aging have given raise to the compensation-related utilization of neural circuits hypothesis (CRUNCH). In this review, we survey both behavioral, functional, and structural changes relevant to inhibitory control in aging. In line with CRUNCH, we found that older adults engage additional brain regions than younger adults when performing the same cognitive task, to compensate for declining brain structures and functions. Moreover, we propose CRUNCH could well take functional inhibitory deficits in older adults into account. Finally, we provide three sensible future research directions.

## Introduction

Inhibitory control is the ability to suppress actions when they are unlikely to accomplish valuable results. Indeed, we are usually controlled by the older habits we have and the external stimuli in the environment more than we realize (Diamond, 2013). Having the ability to inhibit irrelevant thoughts and behaviors makes it possible to change and choose (Diamond, 2013). In experimental psychology, inhibitory control ability could be measured using a wide variety of paradigms, such as the Stroop task, Simon task, Flanker task, antisaccade tasks, delay-of-gratification task, go/no-go task, and stop-signal task (SST).

Contemporary cognitive neuroscience research has also shed light upon the neural basis that supports this fundamental cognitive process. For example, several pioneering studies (e.g., Rubia et al., 2001a,b; Aron et al., 2003, 2004) using functional magnetic resonance imaging (fMRI) on healthy participants found heightened activities in the right inferior frontal gyrus (rIFG) and the anterior insula (aIns). Thus, they concluded that inhibitory control is predominately dedicated to the rIFG/aIns. However, subsequent studies argued that rIFG/aIns is involved in other sub-processes required in inhibition; inhibitory control should be considered as one of the border classes of cognitive control, which is supported by the fronto-parietal network like the multiple-demand (MD) system (Duncan, 2010).

Although the neural basis of inhibitory control is widely investigated, recently there is growing interest in the age-related behavioral and brain alterations related to inhibition. For example, SST makes it possible to determine the stop-signal reaction time (SSRT), which is a measurement of latency of the processes that underpins the behavioral inhibition. Several earlier studies suggest that older adults have worse performance in SST compared to younger adults (Kramer et al., 1994; Williams et al., 1999; Bedard et al., 2002). Moreover, these deficits relate to changes in brain structures and activities. For example, reduction in white matter tracts between the right inferior frontal cortex (rIFC), presupplementary motor area (pre-SMA), and subthalamic nucleus (STN) could predicts this decline in inhibitory control abilities (Coxon et al., 2012).

Several commonalities between these studies were that most studies reported decreased activities in the core inhibitory control region but increased activities in additional brain regions in aging adults. These findings could be explained by the compensation-related utilization of neural circuits hypothesis (CRUNCH), which suggests that older adults engage additional brain regions than younger adults when performing the same cognitive task, to compensate for declining brain structures and functions.

There are several potential models of cognitive aging in the literature, which includes the dedifferentiate hypothesis, PASA model, HAROLD, and ELSA. Specifically, the dedifferentiation hypothesis proposes that different cognitive operations become increasingly reliant on common neural systems with aging. The PASA (posterior-anterior shift in aging) model suggests that age-related reduction in occipital activities accompanied with increased frontal activities is associated with cognitive declines due to aging (e.g., Grady et al., 1994; Davis et al., 2008). The HAROLD model (hemispheric asymmetry reduction in older adults) states that prefrontal activities recruited by cognitive tasks are less lateralized in older adults comparing to younger adults (Cabeza, 2002). These results were replicated using episodic, semantic, operational memory, inhibitory control and perception tasks, thus being able to state that it is a generalized and non-domain-specific phenomenon. Therefore, older adults recruit multiple regions of the brain to perform a task that *a priori* required the activity of only one hemisphere, thus favoring an increase in the success rate. In the end, the ELSA model (early-to-late shift in aging) places particular emphasis on the fact that neurocognitive differences found in activation patterns also lead to a differential temporal pattern with age, which seems to show that older adults resort to action control strategies that compensate for deficits (Grandi and Tirapu Ustárroz, 2017).

**FIGURE 1:**
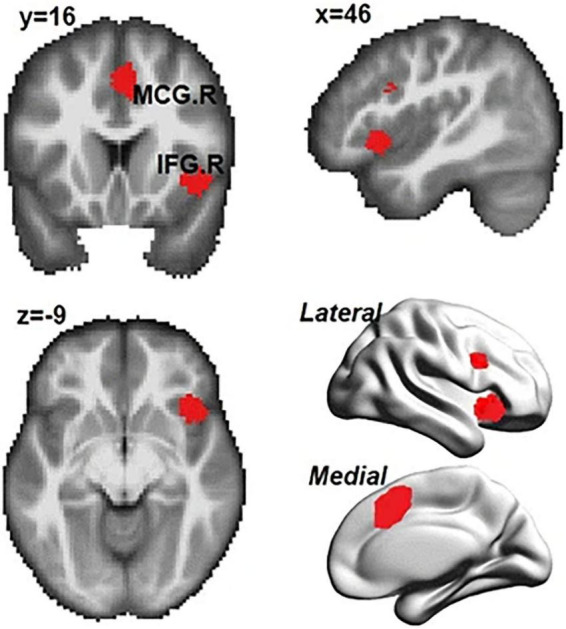
Common regions activated in subprocesses of inhibition (i.e., interference resolution, action withholding, and action cancelation). IFG.R, right inferior frontal gyrus; MCG.R, right median cingulate and paracingulate gyri. Adopted from Zhang et al. (2017) under CC-BY.

Over the several decades, predominant theory suggests that the region within the rIFG/aIns in the human brain is dedicated for inhibitory control. However, there is growing evidence suggesting that inhibitory control should be one of the broader classes that is supported by the same sets of frontoparietal networks [see Hampshire and Sharp (2015) for a review]. In a recent meta-analysis, Zhang et al. (2017) reanalyzed data from 225 studies consisting of 323 experiments to investigate the common and distinct cognitive processes for response inhibition. Zhang et al. (2017) extracted activations coordinates for each subcategory using multilevel kernel density analysis (MKDA). Moreover, Zhang et al. (2017) mapped the extracted brain activation patterns onto functional networks to derive the common and distinct neural correlates for these sub-processes. Zhang et al. (2017) found consistent activations in the right hemisphere regions (e.g., the inferior frontal gyrus, insula, median cingulate, and paracingulate gyri) and the superior parietal gyrus was commonly activated across the sub-cognitive processes studied (Figure 1). The results showed that the frontal-parietal networks and the ventral attentional network are the core brain systems that were commonly recruited in different sub-processes of inhibition. Subtraction analyses revealed different neural substrates involved in interference resolution, action withholding, and action cancelation. Specifically, there were strong activities in the ventral attention network for interference resolution than action inhibition. By contrast, action withholding/cancelation mainly engaged the fronto-striatal circuit whereas interference resolution did not. Zhang et al. (2017) concluded that inhibitory control is a multidimensional cognitive process that engages different brain regions for achieving optimal performance.

The remaining part of this review is organized as follows: first we introduce the CRUNCH. After that, we shed lights upon the behavioral and neural alteration in older adults relating to inhibitory control and evaluate the evidence and counter evidence for CRUNCH in inhibitory control ability; we propose that CRUNCH could well explain the functional changes of inhibitory control abilities in aging. Morever, we also investigate on inhibitory control related neurological disease in aging. Finally, we discuss the potential limitations of this review and outline some sensible directions for future research.

## The Compensation-Related Utilization of Neural Circuits Hypothesis

Before the invention of neuroimaging techniques, traditional behavioral methods and logic in cognitive aging factors view the minimal age differences in cognitive performance between older adults and younger adults are explained by the minimal alteration in neural substrates involved. However, subsequent neuroimaging studies have proven this account is erroneous [see Rueter-Lorenz and Cappell (2008) for a review]. First proposed by Rueter-Lorenz and Cappell (2008), the CRUNCH suggests that older adults engage additional brain regions than younger adults when performing the same cognitive task, to compensate for declining brain structures and functions. Moreover, when individual reaches a critical point (the “CRUNCH” point), where task difficulty exceeds their resources (usually at higher task demands), the brain activities according to fMRI and behavioral performance decline. Indeed, consistent with the CRUNCH, older adults show increased fMRI activities at lower task demands (compensatory fMRI over-activation), comparing to younger adults who have increased fMRI activity at high task demands (Reuter-Lorenz and Cappell, 2008; Soloveva et al., 2018). At the intermediate levels of task demands, older adults reach the ‘CRUNCH’ point because the compensatory mechanism breaks down, which results in decreased fMRI activities in older adults (Soloveva et al., 2018).

## Inhibitory Control Declines in Older Adults

Inspired by research in impaired working memory and the ability to restrain impulses, inhibitory control deficit is proposed to be one of the most essential causes of cognitive declines (Hasher and Zacks, 1988; Zacks et al., 2000). The behavioral evidence suggesting the decline of inhibitory control can be found in several inhibitory control tasks we mentioned, such as the Simon task (West and Alain, 2000; Van der Lubbe and Verleger, 2002; Kubo-Kawai and Kawai, 2010; Maylor et al., 2011) or the SST (Williams et al., 1999; Andrés et al., 2008; Hu et al., 2012), and the go/no-go task (e.g., Rush et al., 2006; Vallesi and Stuss, 2010; Grandjean and Collette, 2011).

A recent study recruited 49 healthy participants in a fMRI session and asked participants to perform a go/no-go, a Simon, and a SST. Moreover, these tasks are qualitatively different from each other, especially in terms of response related complexity. For instance, a simple go/no-go task only requires a unimanual response, whereas, Simon and SST could have two alternative responses thus require bimanual responses. These differences contribute quantitatively to inhibitory control demands, and therefore inhibitory control load. The logic is that preparing a single response as in the simple go/no-go task is much easier than in tasks that require choosing between two or more alternatives as in the Simon’s task and SST. Moreover, canceling an ongoing motor actions as required in the SST could further increase the inhibitory control demands comparing to suppressing motor response tendencies. Sebastian et al. (2013) performed regression analyses by taking age as the predictor to identify the relationship between activation patterns and changes in aging. The results showed a different effect of age on sub-processes of response inhibition. Specifically, in the go/no-go tasks, increased activations of the core inhibitory control network [i.e., the inferior frontal gyrus (IFG), right middle frontal gyrus (MFG), pre-supplementary motor area (pre-SMA), and basal ganglia] and additional parietal areas. In the Simon task, aging was associated with activities in additional inhibitory control regions [i.e., the left PFC, attentional circuits (fronto-parietal network), and regions that control for higher task-set maintenance (cingulo-opercular network)]. However, in the SST, aging was associated with decreased activation in core inhibitory control regions. This suggests that older adults increasingly recruit the inhibitory network and additional brain regions with increasing loads. However, if inhibitory load exceeds compensatory capacity, performance declines along with decreasing activation.

Hsieh and Lin (2017) used SST to examine the age-related inhibitory control performance in a recent electroencephalogram (EEG) study. Hsieh and Lin (2017) recruited 24 adults aged between 20 and 30 years and 24 older adults aged between 61 and 76 years. The task blocks consisted of pure choice reaction-time blocks, global stop-signal blocks with an auditory stop signal, and selective stop-signal blocks with valid and invalid stop signals, which vary in demands. Hsieh and Lin (2017) found that there was a decline in reactive inhibitory control for the older group according to the greater SSRT and reduced amplitude of the P2s peak in both global and selective blocks. Moreover, the decreased reactive inhibitory control could be caused by speed-accuracy tradeoffs. Conversely, Hsieh and Lin (2017) found no age-related decline in proactive inhibitory control, which was reflected by slower RTs and reduced P3 peak amplitude during go trials in blocks with stop-signal comparing to these in blocks with pure choice reaction-time tasks, in which the RT and amplitude differences were similar between groups. These results further support the CRUNCH according to evidence such as there were increased activities at the frontal site in older participants, which did not result in any differences in P2 peak amplitudes between electrode sites, and small differences at the Fz site than other sites compared with younger adults. The P3 peak amplitude at the Fz site demonstrated a significant correlation with the RTs of proactive inhibitory control, which showed that larger differences in RT were related to large reductions in P3 peak. Furthermore, Hartmann et al. (2019) used EEG during a practice session with the same inhibitory control task on both a group of young adults (19–40 years old) and older adults (60–76 years old) to see how aging interacted with the effects of inhibitory control training. They studied the older adults to see if the effect of practice on compensatory functional activity could reduce or develop the compensatory activity. The left IFG has been consistently shown to be recruited in older adults to compensate for impairments in the core rIFG/aIns inhibitory region (Nielson et al., 2002; Langenecker and Nielson, 2003; Hartmann et al., 2019). Additionally, Hu and Li (2020) demonstrated that aging is linked with decreased reactive response inhibition ability and decreased activities of the right anterior hippocampus contributes to this age-related deficiency. As measured in the SST, proactive control ability seems to be maintained in older people, which could be explained in part by increasing age-related activity in the right posterior hippocampus. Their findings contribute to the corpus of knowledge by connecting the hippocampus to age-related changes in cognitive control (Hu and Li, 2020). More recently, Ziaei et al. (2021) demonstrated that whereas older individuals use the anteriorcingulate network to overcome their intuitive reactions in plausible inferences, the inferior frontal gyrus network leads to greater control over responses in both believable and unreal scenarios. In particular, they used 29 healthy, younger individuals (males and females) and 30 healthy older adults (males and females) in which they were asked to judge the logical validity of findings. Unlike younger individuals, older adults used a large-scale network during the conclusion stage, encompassing the anterior cingulate cortex and the inferior frontal gyrus. Thus, this study seemed to support the CRUNCH.

## Structural Changes Underlying Inhibitory Control

Functional imaging studies indeed provide insights into the alterations in brain activation patterns during aging. Moreover, previous studies have found reduction in white matter tracts between the rIFC, pre-SMA, and subthalamic nucleus (STN) could predict declines in inhibitory control abilities (Coxon et al., 2012). The association between preSMA–STN tract connection strength and stopping ability is of particular interests in light of an fMRI study that have demonstrated reduced STN activations during task switching with an inhibition component (Coxon et al., 2010). Less integrated pathway between preSMA and STN may lead to “structural disconnection” (O’Sullivan et al., 2001) and the loss of the ability to functionally modulate STN in older age. However, it is unknown if the structural and functional changes happen at the same time and whether reduced gray matter volume (GMV) could predict deficits in inhibitory control. A more recent study addressed these questions in a cohort of 149 participants using both functional and structural magnetic resonance imaging (MRI; Hu et al., 2018) with the SST. Specifically, stop signal reaction time was used to evaluate the inhibitory control performance in each individual participant. Results demonstrated that aging was associated with longer SSRT in participants. Reduction in GMV and decreased activities of many cortical and subcortical areas was associated with aging. Moreover, age-related impairments in inhibitory control according to the SST were related to both distinct functional and anatomical cerebral changes. Specifically, voxel-based morphometry (VBM) showed that reductions in GMV in the right dorsolateral prefrontal cortex (DLPFC), the caudate head and bilateral insular due to aging were associated with longer SSRT. Aging was also associated with lower activation levels in the medial and inferior frontal cortex in the contrast of stop success vs. go success trials. However, only age-related differences in the activity of the medial prefrontal cortex could be explained by the reduction in GMV, providing limited evidence supporting the structural-functional association. Hu et al. (2018) thus concluded the effects of aging on inhibitory control are modulated by both shared and distinct structural and functional changes during aging. Moreover, there were heightened activities in the right middle occipital gyrus (MOG), which seems to be consistent with the CRUNCH: MOG might compensate for the decreased activities of the reductions of activities in core inhibitory control regions (Hu et al., 2018).

Li et al. (2018) looked at how differences in age and white matter integrity affect Stroop performance (Stroop interference task), as well as whether the effect of age is mediated by white matter integrity in 179 healthy adults from 20 to 80 years old. They discovered a significant inverse correlation between age and the Stroop interference score. In particular, higher Stroop interference score correlated with stronger inhibitory control. On the other hand, white matter tracts showed negative relationships with age and positive relationships with Stroop interference score. They showed that age had a substantial indirect influence on Stroop interference score via the fractional anisotropy (FA) of the left corticospinal tract and the right inferior longitudinal fasciculus. Their findings highlight that the Stroop inhibitory performance is supported by the role of several major white matter tracts and also, they demonstrated that the lower white matter integrity of certain tracts was also identified as a contributor to the loss in inhibitory control capacity associated with the Stroop task in older age.

## Evidence Against The Crunch

However, there is some other counter evidence that argues against the CRUNCH (Turner and Spreng, 2012). For instance, a recent meta-analysis used likelihood estimation analysis on previous imaging studies across several different types of inhibitory control tasks. Turner and Spreng (2012) found that age-related hypoactivations were associated with inhibitory control abilities, whereas there was declined activation in the visual cortical areas. The increased brain activation pattern is termed as the “younger plus” pattern. It is possible that unlike younger adults, these brain regions have increased activities in order to overcome their own declining efficiency.

Additionally, Hu et al. (2014) studied cerebral grey matter (GM) volumes and fractional amplitude of low-frequency fluctuation (fALFF) of blood oxygenation changes related to age in 111 healthy people ranging in age from 18 to 72 years old. They discovered that GM volumes decreased with age in the prefrontal/frontal regions, bilateral insula, and left inferior parietal lobule (IPL). Hence, these findings suggested that these areas are structurally vulnerable to aging. They also found that fALFF was negatively correlated with age, above all in the supplementary motor area (SMA), anterior cingulate cortex, pre-SMA, bilateral dorsal lateral prefrontal cortex (DLPFC), right IPL, and posterior cingulate cortex. Taken together, these findings indicated that neural activities in these areas during cognitive performance decline with age. Furthermore, their results are in line with previous findings of the fact that the activity of the pre-supplementary motor region reflects inhibitory control (Li et al., 2006; Chao et al., 2009; Duann et al., 2009). An additional result of Hu et al. (2014) research was that differences in cognitive functioning over aging may be explained by anatomical and functional changes in the pre-SMA. However, these findings did not show additional regions active during the inhibitory control task. Recently, Le et al. (2020) used fMRI data from 72 individuals (aged 21–74) conducting a reward go/no-go task to investigate how aging affects incentive-directed action and inhibition of action differently. Their findings revealed that as age increased, the response time (RT) in rewarded (vs. control) go trials increased. They found that anterior insula, middle frontal gyrus, and rostral anterior cingulate cortex activity decreased with age. They hypothesized that aging affects shared brain substrates that govern both action and inhibition. Activities in these brain areas may reflect different brain alterations.

## Inhibitory Control Related Neurological Disease in Aging

There are notable pieces of evidence detectable from using CRUNCH quantitative model of compensation to characterize compensation in neurological diseases (Klöppel et al., 2009, 2015; Gray et al., 2013; Georgiou-Karistianis et al., 2014; Malejko et al., 2014; Soloveva et al., 2018, 2020; Poudel et al., 2019). For example, premanifest Huntington’s disease (pre-HD) individuals typically show increased task-related fMRI, which were thought to reflect compensatory strategies. In line with the CRUNCH prediction, Soloveva et al. (2020) found that pre-HD individuals show decreased fMRI activities in left intraparietal sulcus at high memory load (visuospatial working memory task), compared to healthy controls who show increased fMRI activities in left intraparietal sulcus at high memory demand. In contrast to the other CRUNCH prediction, the pre-HD group did not show compensatory increase in fMRI activities at lower levels of memory loads in left intraparietal sulcus. Their findings provided partial support for the validity of CRUNCH in pre-HD. In pre-HD, consistent with compensation during a working memory task, the performance-related activities in the right parietal cortex increased as atrophy increased. Similarly, increased functional connectivity between the right DLPFC and a left hemisphere network in the resting-state predicted better cognitive performance as atrophy increased. Such patterns cannot be found in the lift hemisphere or in the motor tasks (Klöppel et al., 2015).

Importantly, Gray et al. (2013) observed that symptomatic-HD (symp-HD) have greater prefrontal functional responses comparing to controls and pre-HD, including larger activations and deactivations in response to cognitive challenges, which is consistent with the CRUNCH. Moreover, they also found that reduced prefrontal responsivity in symp-HD is related with increased neuropsychiatric disturbance within domains including disinhibition, pathological impulses, executive dysfunction, and depression. Prefrontal activities during inhibitory attentional control usefully characterized cognitive and neuropsychiatric status in symp-HD. Thus, they suggested that functional integrity of compensatory prefrontal responses may provide a useful marker for treatments which aim to sustain cognitive function and delay executive and neuropsychiatric disturbance (Gray et al., 2013).

However, pre- and symp-HD individuals demonstrated increased neural activities in the context of intact behavioral performance (Klöppel et al., 2009; Georgiou-Karistianis et al., 2014; Malejko et al., 2014; Poudel et al., 2019), which is indicative of attempted compensation. Thus, fMRI overactivities in HD may in fact reflect successful compensation even in the absence of improved behavioral performance (Zarahn et al., 2007), which can be explained by the neurodegenerative nature of HD, associated with widespread neuropathological processes. Soloveva et al. (2018) postulated that the CRUNCH is the most relevant model based on existing HD research and thus should be regarded as a possible starting point as part of developing a HD-specific model of compensation. On the other hand, PASA or HAROLD do not seem to has relevance to the HD picture.

## Future Research Directions

Inhibitory control is an important ability declining with aging but much less investigated. We propose there are several directions for future research: (1) It is important for future research to consider other cognitive demands in inhibitory tasks, for example, Simmonds et al. (2008) have found hyperactivation in the fronto-parietal network in the go/no-go tasks with high working memory demands. It is possible that older adults may have deficits in other cognitive abilities that are important in successfully performing inhibitory control tasks, which affect the performance of the inhibitory control tasks, rather than directly having inhibition control impairments. (2) From a translational-clinical perspective, it has been proposed that patients with degenerative neurological conditions are more impaired in inhibitory control than normal aging adults. Thus, it would be important for future research to investigate whether inhibitory control tasks could be utilized as biomarkers for various neurological conditions during aging and how it can affect functional outcomes. (3) Longitudinal studies may be necessary for future endeavors as it can rule out the possibility of pre-existing conditions.

## Conclusion

The present review focused on the neural basis of inhibitory control and compared the behavioral, structural, and functional changes during aging. Moreover, we evaluated evidence both for and against the CRUNCH and conclude that CRUNCH is useful to take inhibitory control declines in older adults into account. Older adults reach an asymptote in both behavior and brain activation at lower levels comparing to younger adults (Schneider-Garces et al., 2009). The individualized span analysis in Schneider-Garces et al. (2009) provided an even stranger quantitative support for the CRUNCH, which showed that differences in span across individuals regardless of age can fully account for the differences in the brain-activation-by-memory-load function between young and older people. The curves for young and older adults are identical after these differences are taken into account. Therefore, as this difference can be explained by relative task difficulty, no special mechanisms are needed to account for the different patterns of brain activities between young and older adults. Evidence supporting the CRUNCH model can also be found in neurological disease in aging (Klöppel et al., 2009, 2015; Gray et al., 2013; Georgiou-Karistianis et al., 2014; Malejko et al., 2014; Soloveva et al., 2018, 2020; Poudel et al., 2019).

However, experimental evidence against CRUNCH is provided by Jamadar (2020), who did not find evidence for differential load- dependent changes in fMRI activities in older compared to young adults. It could be argued that the highest level of difficulty was not sufficiently difficult to induce a CRUNCH effect (Jamadar, 2020). One would argue that sample size was insufficient to detect this effect. Furthermore, the results in each region of interest (ROI), except the visual cortex, which showed opposite effects of what would be predicted by the CRUNCH, suggesting that the modest sample size does not explain the failure to replicate the CRUNCH predictions. It is surprising that although the CRUNCH model is highly influential in the literature, only a few studies have directly tested its hypotheses, and the existing studies are restricted only to memory. Despite these pieces of evidence, the CRUNCH model has been influential because it represents an important step toward quantifiable and falsifiable models of cognitive compensation in aging. Thus, it is important for future studies to conduct larger and better controlled studies to test the CRUNCH.

Our work provided a novel perspective on the brain activation patterns in inhibitory control tasks during aging and foundations for future neuroscientific research. Our conclusion may be biased by the neuroimaging methods and the preexisting conditions given currently available studies are cross-sectional rather than longitudinal according to our knowledge. Future studies should (1) consider other cognitive demands in inhibitory tasks, (2) investigate whether inhibitory control tasks could be utilized as biomarkers for various neurological conditions during aging, and (3) conduct longitudinal research which rules out the possibility of pre-existing conditions.

## Author Contributions

WK: conceptualization, writing – original draft and review and editing, visualization, and funding acquisition. JW and AM: writing – review and editing. All authors contributed to the article and approved the submitted version.

## Conflict of Interest

The authors declare that the research was conducted in the absence of any commercial or financial relationships that could be construed as a potential conflict of interest.

## Publisher’s Note

All claims expressed in this article are solely those of the authors and do not necessarily represent those of their affiliated organizations, or those of the publisher, the editors and the reviewers. Any product that may be evaluated in this article, or claim that may be made by its manufacturer, is not guaranteed or endorsed by the publisher.

## References

[B1] Andrés P. Guerrini C. Phillips L. H. Perfect T. J. (2008). Differential effects of aging on executive and automatic inhibition. *Dev. Neuropsychol.* 33 101–123. 10.1080/87565640701884212 18443972

[B2] Aron A. R. Fletcher P. C. Bullmore E. T. Sahakian B. J. Robbins T. W. (2003). Stop-signal inhibition disrupted by damage to right inferior frontal gyrus in humans. *Nat. Neurosci.* 6 115–116. 10.1038/nn1003 12536210

[B3] Aron A. R. Robbins T. W. Poldrack R. A. (2004). Inhibition and the right inferior frontal cortex. *Trends Cognit. Sci.* 8 170–177. 15050513 10.1016/j.tics.2004.02.010

[B4] Bedard A. C. Nichols S. Barbosa J. A. Schachar R. Logan G. D. Tannock R. (2002). The development of selective inhibitory control across the life span. *Dev. Neuropsychol.* 21 93–111. 10.1207/S15326942DN2101_5 12058837

[B5] Cabeza R. (2002). Hemispheric asymmetry reduction in older adults: the HAROLD model. *Psychol. Aging* 17 85–100. 10.1037/0882-7974.17.1.85 11931290

[B6] Rueter-Lorenz P. Cappell K. A. (2008). Neurocognitive aging and the compensation hypothesis. *Curr. Direct. Psychol. Sci.* 17 177–182.

[B7] Chao H. H. Luo X. Chang J. L. Li C. S. (2009). Activation of the pre-supplementary motor area but not inferior prefrontal cortex in association with short stop signal reaction time – an intra-subject analysis. *BMC Neurosci.* 10:75. 10.1186/1471-2202-10-75 19602259 PMC2719646

[B8] Coxon J. P. Goble D. J. Van Impe A. De Vos J. Wenderoth N. Swinnen S. P. (2010). Reduced basal ganglia function when elderly switch between coordinated movement patterns. *Cereb. Cortex* 20 2368–2379. 20080932 10.1093/cercor/bhp306

[B9] Coxon J. P. Goble D. J. Leunissen I. Van Impe A. Wenderoth N. Swinnen S. P. (2016). Functional brain activation associated with inhibitory control deficits in older adults. *Cereb. Cortex* 26 12–22. 10.1093/cercor/bhu165 25085883

[B10] Coxon J. P. Van Impe A. Wenderoth N. Swinnen S. P. (2012). Aging and inhibitory control of action: cortico-subthalamic connection strength predicts stopping performance. *J. Neurosci.* 32 8401–8412. 10.1523/JNEUROSCI.6360-11.2012 22699920 PMC6703643

[B11] Davis S. W. Dennis N. A. Daselaar S. M. Fleck M. S. Cabeza R. (2008). Que PASA? The posterior-anterior shift in aging. *Cereb. Cortex* 18 1201–1209. 10.1093/cercor/bhm155 17925295 PMC2760260

[B12] Diamond A. (2013). Executive functions. *Annu. Rev. Psychol.* 64 135–168. 23020641 10.1146/annurev-psych-113011-143750 PMC4084861

[B13] Duann J. R. Ide J. S. Luo X. Li C. S. (2009). Functional connectivity delineates distinct roles of the inferior frontal cortex and presupplementary motor area in stop signal inhibition. *J. Neurosci.* 29 10171–10179. 19675251 10.1523/JNEUROSCI.1300-09.2009 PMC2769086

[B14] Duncan J. (2010). The multiple-demand (MD) system of the primate brain: mental programs for intelligent behaviour. *Trends Cognit. Sci.* 14 172–179. 10.1016/j.tics.2010.01.004 20171926

[B15] Georgiou-Karistianis N. Long J. D. Lourens S. G. Stout J. C. Mills J. A. Paulsen J. S. (2014). Predict-Hd Investigators and Coordinators Of The Huntington Study Group (Hsg). (2014). Movement sequencing in Huntington disease. *World J. Biol. Psychiatry* 15 459–471. 10.3109/15622975.2014.895042 24678867 PMC4389285

[B16] Grady C. L. Maisog J. M. Horwitz B. Ungerleider L. G. Mentis M. J. Salerno J. A. (1994). Age-related changes in cortical blood flow activation during visual processing of faces and location. *J. Neurosci.* 14(3 Pt 2), 1450–1462. 8126548 10.1523/JNEUROSCI.14-03-01450.1994 PMC6577560

[B17] Grandi F. Tirapu Ustárroz J. (2017). Cognitive neuroscience of aging: explanatory models. *Rev. Esp. Geriatr. Gerontol.* 52 326–331. 28506658 10.1016/j.regg.2017.02.005

[B18] Grandjean J. Collette F. (2011). Influence of response prepotency strength, general working memory resources, and specific working memory load on the ability to inhibit predominant responses: A comparison of younger and elderly participants. *Brain Cognit.* 77 237–247. 10.1016/j.bandc.2011.08.004 21885178

[B19] Gray M. A. Egan G. F. Ando A. Churchyard A. Chua P. Stout J. C. (2013). Prefrontal activity in Huntington’s disease reflects cognitive and neuropsychiatric disturbances: The IMAGE-HD study. *Exp. Neurol.* 239 218–228. 10.1016/j.expneurol.2012.10.020 23123406

[B20] Hampshire A. Sharp D. J. (2015). Contrasting network and modular perspectives on inhibitory control. *Trends Cognit. Sci.* 19 445–452. 10.1016/j.tics.2015.06.006 26160027

[B21] Hartmann L. Wachtl L. de Lucia M. Spierer L. (2019). Practice-induced functional plasticity in inhibitory control interacts with aging. *Brain Cogn.* 132 22–32. 30802731 10.1016/j.bandc.2019.02.004

[B22] Hasher L. Zacks R. T. (1988). “Working memory, comprehension, and aging: a review and a new view,” in *The Psychology of Learning and Motivation: Advances in Research and Theory*, Vol. 22, ed. Bower G. H. (San Diego, CA: Academic Press), 193–225.

[B23] Hsieh S. Lin Y. C. (2017). Stopping ability in younger and older adults: Behavioral and event-related potential. *Cognit. Affect. Behav. Neurosci.* 17 348–363. 10.3758/s13415-016-0483-7 27896714

[B24] Hu S. Chao H. H. A. Zhang S. Ide J. S. Li C. S. R. (2014). Changes in cerebral morphometry and amplitude of low-frequency fluctuations of BOLD signals during healthy aging: correlation with inhibitory control. *Brain Struct. Funct.* 219 983–994. 23553547 10.1007/s00429-013-0548-0 PMC3760988

[B25] Hu S. Chao H. H. A. Winkler A. D. Li C. S. R. (2012). The effects of age on cerebral activations: internally versus externally driven processes. *Front. Aging Neurosci.* 4:4. 10.3389/fnagi.2012.00004 22536185 PMC3334814

[B26] Hu S. Ide J. S. Chao H. H. Castagna B. Fischer K. A. Zhang S. (2018). Structural and functional cerebral bases of diminished inhibitory control during healthy aging. *Hum. Brain Mapp.* 39 5085–5096. 10.1002/hbm.24347 30113124 PMC6287913

[B27] Hu S. Li C. R. (2020). Age-related structural and functional changes of the hippocampus and the relationship with inhibitory control. *Brain Sci.* 10:1013. 10.3390/brainsci10121013 33352718 PMC7766783

[B28] Jamadar S. D. (2020). The CRUNCH model does not account for load-dependent changes in visuospatial working memory in older adults. *Neuropsychologia* 142:107446. 10.1016/j.neuropsychologia.2020.107446 32234498

[B29] Klöppel S. Gregory S. Scheller E. Minkova L. Razi A. Durr A. (2015). Compensation in Preclinical Huntington’s Disease: Evidence From the Track-On HD Study. *EBioMed.* 2 1420–1429. 10.1016/j.ebiom.2015.08.002 PMC4634199 26629536

[B30] Klöppel S. Henley S. M. Hobbs N. Z. Wolf R. C. Kassubek J. Tabrizi S. J. (2009). Magnetic resonance imaging of Huntington’s disease: preparing for clinical trials. *Neuroscience* 164 205–219. 10.1016/j.neuroscience.2009.01.045 19409230 PMC2771270

[B31] Kramer A. F. Humphrey D. G. Larish J. F. Logan G. D. (1994). Aging and inhibition: beyond a unitary view of inhibitory processing in attention. *Psychol. Aging* 9:491. 7893421

[B32] Kubo-Kawai N. Kawai N. (2010). Elimination of the enhanced Simon effect for older adults in a three-choice situation: Ageing and the Simon effect in a go/no-go Simon task. *Quart. J. Exp. Psychol.* 63 452–464. 10.1080/17470210902990829 19575334

[B33] Langenecker S. A. Nielson K. A. (2003). Frontal recruitment during response inhibition in older adults replicated with fMRI. *Neuroimage* 20 1384–1392. 14568507 10.1016/S1053-8119(03)00372-0

[B34] Le T. M. Chao H. Levy I. Li C. S. R. (2020). Age-related changes in the neural processes of reward-directed action and inhibition of action. *Front. Psychol.* 11:1121. 10.3389/fpsyg.2020.01121 32587547 PMC7298110

[B35] Li S. C. Lindenberger U. Sikström S. (2006). Aging cognition: from neuromodulation to representation. *Trends Cognit. Sci.* 5 479–486. 10.1016/s1364-6613(00)01769-1 11684480

[B36] Li P. Tsapanou A. Qolamreza R. R. Gazes Y. (2018). White matter integrity mediates decline in age-related inhibitory control. Behav. Brain Res. 339, 249–254. 10.1016/j.bbr.2017.11.005 29126930 PMC5729101

[B37] Malejko K. Weydt P. Süßmuth S. D. Grön G. Landwehrmeyer B. G. Abler B. (2014). Prodromal Huntington disease as a model for functional compensation of early neurodegeneration. *PLoS One* 9:e114569. 10.1371/journal.pone.0114569 25541992 PMC4277279

[B38] Maylor E. A. Birak K. S. Schlaghecken F. (2011). Inhibitory motor control in old age: evidence for de-automatization? *Front. Psychol.* 2:132. 10.3389/fpsyg.2011.00132 21734899 PMC3122077

[B39] Nielson K. A. Langenecker S. A. Garavan H. (2002). Differences in the functional neuroanatomy of inhibitory control across the adult life span. *Psychol. Aging* 17 56–71. 10.1037//0882-7974.17.1.56 11931287

[B40] O’Sullivan M. Jones D. K. Summers P. E. Morris R. G. Williams S. C. Markus H. S. (2001). Evidence for cortical “disconnection” as a mechanism of age-related cognitive decline. *Neurology* 57 632–638. 10.1212/wnl.57.4.632 11524471

[B41] Poudel G. R. Harding I. H. Egan G. F. Georgiou-Karistianis N. (2019). Network spread determines severity of degeneration and disconnection in Huntington’s disease. *Hum. Brain Mapp.* 40 4192–4201. 10.1002/hbm.24695 31187915 PMC6865500

[B42] Prakash R. S. Erickson K. I. Colcombe S. J. Kim J. S. Voss M. W. Kramer A. F. (2009). Age-related differences in the involvement of the prefrontal cortex in attentional control. *Brain Cognit.* 71 328–335. 19699019 10.1016/j.bandc.2009.07.005 PMC2783271

[B43] Reuter-Lorenz P. A. Cappell K. A. (2008). Neurocognitive aging and the compensation hypothesis. *Curr. Dir. Psychol. Sci.* 17, 17–182.

[B44] Rubia K. Russell T. Overmeyer S. Brammer M. J. Bullmore E. T. Sharma T. (2001a). Mapping motor inhibition: conjunctive brain activations across different versions of go/no-go and stop tasks. *Neuroimage* 13 250–261. 10.1006/nimg.2000.0685 11162266

[B45] Rubia K. Smith A. Lidzba K. Toone B. Simmons A. Williams S. C. (2001b). Neural substrates of successful versus unsuccessful stopping in a cognitively challenging event related stop task. *Neuroimage* 6:351. 10.1016/s1053-8119(01)91694-5

[B46] Rush B. K. Barch D. M. Braver T. S. (2006). Accounting for cognitive aging: context processing, inhibition or processing speed? *Aging Neuropsychol. Cognit.* 13 588–610. 10.1080/13825580600680703 16887791

[B47] Schneider-Garces N. J. Gordon B. A. Brumback-Peltz C. R. Shin E. Lee Y. Sutton B. P. (2009). Span, CRUNCH, and Beyond: Working Memory Capacity and the Aging Brain. *J. Cogn. Neurosci.* 22 655–669. 10.1162/jocn.2009.21230 19320550 PMC3666347

[B48] Sebastian A. Baldermann C. Feige B. Katzev M. Scheller E. Hellwig B. (2013). Differential effects of age on subcomponents of response inhibition. *Neurobiol. Aging* 34 2183–2193. 23591131 10.1016/j.neurobiolaging.2013.03.013

[B49] Simmonds D. J. Pekar J. J. Mostofsky S. H. (2008). Meta-analysis of Go/No-go tasks demonstrating that fMRI activation associated with response inhibition is task-dependent. *Neuropsychologia* 46 224–232. 10.1016/j.neuropsychologia.2007.07.015 17850833 PMC2327217

[B50] Soloveva M. V. Jamadar S. D. Hughes M. Velakoulis D. Poudel G. Georgiou-Karistianis N. (2020). Brain compensation during response inhibition in premanifest Huntington’s disease. *Brain Cogn.* 141:105560. 10.1016/j.bandc.2020.105560 32179366

[B51] Soloveva M. V. Jamadar S. D. Poudel G. (2018). A critical review of brain and cognitive reserve in Huntington’s disease. *Neurosci. Biobehav. Rev.* 88 155–169. 10.1016/j.neubiorev.2018.03.003 29535068

[B52] Turner G. R. Spreng R. N. (2012). Executive functions and neurocognitive aging: dissociable patterns of brain activity. *Neurobiol. Aging* 33 826.e1–826.e13. 10.1016/j.neurobiolaging.2011.06.005 21791362

[B53] Vallesi A. Stuss D. T. (2010). Excessive sub-threshold motor preparation for non-target stimuli in normal aging. *Neuroimage* 50 1251–1257. 10.1016/j.neuroimage.2010.01.022 20079449

[B54] Vallesi A. McIntosh A. R. Stuss D. T. (2011). Overrecruitment in the aging brain as a function of task demands: evidence for a compensatory view. *J. Cognit. Neurosci.* 23 801–815. 10.1162/jocn.2010.21490 20350184

[B55] Van der Lubbe R. H. Verleger R. (2002). Aging and the Simon task. *Psychophysiology* 39 100–110. 10.1111/1469-8986.3910100 12206290

[B56] West R. Alain C. (2000). Age−related decline in inhibitory control contributes to the increased Stroop effect observed in older adults. *Psychophysiology* 37 179–189. 10731768

[B57] Williams B. R. Ponesse J. S. Schachar R. J. Logan G. D. Tannock R. (1999). Development of inhibitory control across the life span. *Dev. Psychol.* 35:205. 10.1037/0012-1649.35.1.205 9923475

[B58] Zacks R. T. Hasher L. Li K. Z. (2000). “Human memory,” in *Handbook of Aging and Cognition*, 2nd Edn, eds Salthouse T. A. Craik F. I. M. (Mahwah, NJ: Lawrence Erlbaum), 293–357.

[B59] Zarahn E. Rakitin B. Abela D. Flynn J. Stern Y. (2007). Age-related changes in brain activation during a delayed item recognition task. *Neurobiol. Aging* 28 784–798. 10.1016/j.neurobiolaging.2006.03.002 16621168

[B60] Ziaei M. Bonyadi M. R. Reutens D. C. (2021). Age-related differences in structural and functional prefrontal networks during a logical reasoning task. *Brain Imaging Behav.* 15 1085–1102. 32556885 10.1007/s11682-020-00315-5

[B61] Zhang R. Geng X. Lee T. M. (2017). Large-scale functional neural network correlates of response inhibition: an fMRI meta-analysis. *Brain Struct. Funct.* 222 3973–3990. 10.1007/s00429-017-1443-x 28551777 PMC5686258

